# Non‐eosinophilic asthma in nonsteroidal anti‐inflammatory drug exacerbated respiratory disease

**DOI:** 10.1002/clt2.12235

**Published:** 2023-03-13

**Authors:** Lucyna Mastalerz, Natalia Celejewska‐Wójcik, Adam Ćmiel, Krzysztof Wójcik, Joanna Szaleniec, Karolina Hydzik‐Sobocińska, Jerzy Tomik, Marek Sanak

**Affiliations:** ^1^ 2nd Department of Internal Medicine Jagiellonian University Medical College Krakow Poland; ^2^ Department of Applied Mathematics AGH University of Science and Technology Krakow Poland; ^3^ Department of Otolaryngology Faculty of Medicine Jagiellonian University Medical College Krakow Poland

**Keywords:** aspirin hypersensitivity, cluster analysis, inflammatory phenotypes, non‐eosinophilic asthma

## Abstract

**Background:**

The cellular inflammatory pattern of nonsteroidal anti‐inflammatory drug–exacerbated respiratory disease (N‐ERD) is heterogeneous. However, data on the heterogeneity of non‐eosinophilic asthma (NEA) with aspirin hypersensitivity are scanty. By examination of N‐ERD patients based on clinical data and eicosanoid biomarkers we aimed to identify NEA endotypes potentially guiding clinical management.

**Methods:**

Induced sputum was collected from patients with N‐ERD. Sixty six patients (49.6% of 133 N‐ERD) with NEA were included in the hierarchical cluster analysis based on clinical and laboratory data. The quality of clustering was evaluated using internal cluster validation with different indices and a practical decision tree was proposed to simplify stratification of patients.

**Results:**

The most frequent NEA pattern was paucigranulocytic (PGA; 75.8%), remaining was neutrophilic asthma (NA; 24.2%). Four clusters were identified. Cluster #3 included the highest number of NEA patients (37.9%) with severe asthma and PGA pattern (96.0%). Cluster #1 (24.2%) included severe only asthma, with a higher prevalence of NA (50%). Cluster #2 (25.8%) comprised well‐controlled mild or severe asthma (PGA; 76.5%). Cluster #4 contained only 12.1% patients with well‐controlled moderate asthma (PGA; 62.5%). Sputum prostaglandin D_2_ levels distinguished cluster #1 from the remaining clusters with an area under the curve of 0.94.

**Conclusions:**

Among identified four NEA subtypes, clusters #3 and #1 represented N‐ERD patients with severe asthma but a different inflammatory signatures. All the clusters were discriminated by sputum PGD_2_ levels, asthma severity, and age of patients. The heterogeneity of non‐eosinophilic N‐ERD suggests a need for novel targeted interventions.

## 1 INTRODUCTION

Nonsteroidal anti‐inflammatory drug (NSAID)–exacerbated respiratory disease (N‐ERD) is characterized by asthma with symptoms exacerbated by ingestion of cyclooxygenase‐1 (COX‐1) inhibitors and accompanying chronic rhinosinusitis with nasal polyps (CRSwNP).^1^ Despite diagnostic criteria based on positive provocation test with NSAID and overproduction of cysteinyl leukotrienes, airway inflammatory patterns of N‐ERD are heterogeneous. An inflammatory pattern based on induced sputum cell counts in N‐ERD encompasses the whole spectrum of eosinophilic, neutrophilic, mixed granulocytic, and paucigranulocytic asthma.^2^, ^3^, ^4^, ^5^, ^6^ Non‐eosinophilic asthma (NEA) corresponds to T2‐low endotype of asthma, by analysis of biomarkers suggesting involvement of Th1 and Th17 helper cells and non‐Th2 mediators such as interleukins IL‐1β, IL‐6, IL‐8, IL‐17 A/F and interferon γ (IFN‐γ).^6^ Heterogeneity of airway inflammatory patterns in patients with N‐ERD is well described.^2^, ^3^, ^4^ It reflects a heterogeneity of a general asthma population.^7^, ^8^, ^9^ Despite a significantly elevated peripheral blood eosinophilia^1^, ^10^ and presence of CRSwNP,^4^ N‐ERD seems not to associate consistently with a severe eosinophilic inflammation of the respiratory mucosa. Based on our previous studies, about half of patients with N‐ERD presented a non‐eosinophilic inflammatory pattern.^2^, ^3^ Moreover, in a severe N‐ERD, neutrophilic or paucigranulocytic inflammation seems as common, as in general population of asthma patients. In adult patients asthma with neutrophilic inflammatory pattern is less responsive to glucocorticoids.^11^ Therefore, a rationale for this study was a scarcity of data on heterogeneity of NEA within N‐ERD patients. We hypothesized that a pattern of non‐eosinophilic airway inflammation in N‐ERD can associate with particular asthma subtypes, different from a classical T2‐high. For this reason we collected data on a well characterized N‐ERD cohort and used a machine‐learning analysis on demographic and laboratory data, including already published predictors of NEA^8^ or non‐eosinophilic CRSwNP.^12^ The latter study on upper airways inflammation^12^ showed, that non‐eosinophilic inflammation was also prevalent in N‐ERD. By a characteristics of non‐eosinophilic inflammatory patterns in N‐ERD one can expect improved management of the disease. Indeed, a suboptimal responses to the anti‐asthmatic treatment of N‐ERD patients could result from a variety of endotypes of the disease.^13^, ^14^ Consequently, a better definition of NEA patients within N‐ERD phenotype offers opportunity to propose therapeutic interventions targeted at more homogenous patients groups.

## 2 MATERIALS AND METHODS

### 2.1 Study group

Participants of this study were enrolled from a prospective database of N‐ERD patients diagnosed and treated at the Department of Internal Medicine, Jagiellonian University Medical College, Krakow, Poland. The analysis included 66 nonsmoking patients with N‐ERD and a non‐eosinophilic inflammatory pattern (49.6% of 133 all N‐ERD patients screened) based on the induced sputum cytology. The diagnosis of aspirin hypersensitivity was confirmed by bronchial or oral aspirin challenge test. Patients were treated with nasal and inhaled corticosteroids (ICSs) and long‐acting β_2_‐agonists for 6 weeks preceding sputum induction. Within 1 year prior enrollment only 5 patients received oral corticosteroids due to exacerbation of asthma but not longer than for 3 weeks. None of the patients used anti‐leukotrienes or biological drugs and no participant had respiratory tract infection or asthma exacerbation during 6 weeks prior sputum sampling. On the day of sputum induction, each participant had a pulmonary function test and forced expiratory volume in the ﬁrst second was always 70% or higher. The characteristics of the study group is presented in Table 1. All patients provided written informed consent to participate in the study. The study was approved by the Jagiellonian University Ethics Committee and the protocol complied with the Declaration of Helsinki.

**TABLE 1:** Characteristics of patients with nonsteroidal anti‐inflammatory drug–exacerbated respiratory disease and with non‐eosinophilic inflammatory pattern in sputum (*n* = 66).

Variable	NEA	Cluster #1	Cluster #2	Cluster #3	Cluster #4	*p* values
	*n* = 66	*n* = 16 (24.2%)	*n* = 17 (25.8%)	*n* = 25 (37.9%)	*n* = 8 (12.1%)	
Age, years	47.5	43.5	35	52	50	<0.001, 0.020,* <0.001**
	(37; 53)	(32.5; 50.5)	(30; 41)	(48; 62)	(42; 52.5)	
Sex, male/female, *n*	20/46	5/11	3/14	8/17	4/4	0.423
BMI, kg/m^2^	26	27	23.7	28	25.5	0.002, 0.005**
	(23.9; 29)	(23.9; 29.4)	(22.6; 25.6)	(26.4; 31.5)	(25.1; 25.8)	
Age of asthma onset, years	35	35	22	42	34.5	<0.001, 0.001,** 0.020***
	(26; 43)	(23; 42.5)	(19; 30)	(35; 48)	(31; 42)	
Asthma duration, years	9	6,5	10	10	10,5	0.989
	(5; 17)	(5; 17.5)	(5; 17)	(6; 14)	(6,5; 17)	
Dose of ICSs, μg/day fluticasone eq.	1000	1000	250	1000	500	<0.001, 0.013,^†^ 0.001**
	(250; 1000)	(500; 1000)	(0; 1000)	(1000; 1000)	(500; 500)	
Moderate‐to‐severe exacerbation in the past year	0.5	2	0	0	0.5	0.021, 0.24^†^
	(0; 2)	(0.5; 3.5)	(0; 1)	(0; 2)	(0; 3)	
FEV_1_, % predicted	95.1	90.2	99.5	90	95.1	0.106
	(87.1; 101.4)	(84.2; 99.3)	(90.3; 101.4)	(81.9; 99.9)	(89.3; 103.7)	
Asthma control, *n* (%)						
Controlled	44 (66.7)	7 (43.8)	16 (94.1)	13 (52)	8 (100)	0.003, 0.009,^‡^ 0.030^§^
Partly controlled	9 (13.6)	5 (31.2)	1 (5.9)	3 (12)	0 (0)	
Uncontrolled	13 (19.7)	4 (25)	0 (0)	9 (36)	0 (0)	
GINA 2022 asthma severity, *n* (%)						
Mild	10 (15.2)	0 (0)	9 (52.9)	1 (3.8)	0 (00)	<0.001, 0.002.^†^ 0.006,** <0.001,^‡^ <0.001^§^
Moderate	8 (12.1)	0 (0)	0 (0)	0 (0)	8 (100)	
Severe	48 (72.7)	16 (100)	8 (47.1)	25 (96.2)	0 (0)	
ACT Score	22	19	24	20	25	0.003, 0.038**
	(18; 25)	(15.5; 25)	(22; 25)	(14; 23)	(23.5; 25)	
CRSwNP (yes/no)	66/0	16/0	17/0	25/0	8/0	
Lund‐Mackay score (CT of the paranasal sinuses)	14 (10; 16)	15 (11; 18)	13 (10; 15)	13 (9; 17)	13.5 (10; 15)	0.680
Blood eosinophils per mm^3^	268	113	320	250	368	0.002, 0.008,^†^ 0.029^‡^
	(190; 398)	(30; 316)	(230; 400)	(220; 360)	(265; 566)	
Skin prick tests (positive/negative)	27/39	3/13	10/7	11/14	3/5	0.130
Total IgE level, IU/mL	61.9	35.5	37.5	90	332	0.024, 0.043^‡^
	(22.3; 240)	(19.9; 62.3)	(26.2; 256)	(19.3; 224)	(77.5; 438.5)	
Sputum NEA patterns, *n* (%)						
Paucigranulocytic	50 (75.8)	8 (50)	13 (76.5)	24 (96)	5 (62.5)	0.006, 0.001,* 0.012^§^
Neutrophilic	16 (24.2)	8 (50)	4 (23.5)	1 (4)	3 (37.5)	
ISS eicosanoids, pg/mL						
LTE_4_	19.5	33.1	28.3	11.9	12.2	0.046
	(7.1; 50.5)	(14.2; 57.6)	(15.1; 112.6)	(6.8; 31.4)	(9.6; 22)	
PGE_2_	58.2	131.2	54.4	37.4	46.5	<0.001, 0.014,^†^ <0.001*
	(34.5; 100.3)	(67.1; 207)	(44; 101.7)	(22.2; 71.2)	(44; 57.3)	
PGD_2_	21.9	81.3	25	15.6	18.1	<0.001, <0.001,^†^ <0.001,* <0.001^‡^
	(13.8; 45.1)	(52,2; 143.4)	(16.7; 36.3)	(8.2; 20.2)	(7.4; 24.9)	
Urinary LTE_4_, pg/mg creatinine	701.9	1354	399	743	382	0.104
	(251; 1336)	(499; 2392)	(192; 698)	(510; 1336)	(167; 1062)	

Values are expressed as number (%) of patients or median (25%; 75%). Post hoc test comparison: *cluster #1 versus cluster #3; **cluster #2 versus cluster #3; ***cluster #2 versus cluster #4; ^†^cluster #1 versus cluster #2; ^‡^cluster #1 versus cluster #4; ^§^cluster #3 versus cluster #4.

Abbreviations: ACT, Asthma Control Test; BMI, body mass index; CRSwNP, chronic rhinosinusitis with nasal polyps; CT, computed tomography; FEV_1_, forced expiratory volume in the first second; ICS, inhaled corticosteroid; IgE, immunoglobulin E; ISS, induced sputum supernatant; LTE_4_, leukotriene four; NEA, non‐eosinophilic asthma; PGD_2_, prostaglandin D_2_; PGE_2_, prostaglandin E_2;_ LTE_4_, leutriene E_4_.

### 2.2 Study design

Patients with N‐ERD subjected to sputum induction were recruited during two prospective studies, the results of the former were already published.^10^, ^13^ The observation period lasted 52 weeks during which the patients received ambulatory care at our center. At the entry to the current study each participant had clinical evaluation including asthma control test, spirometry, and skin prick test. Laboratory investigations included peripheral blood eosinophil count, total immunoglobulin E (IgE) serum level, and urinary leukotriene E_4_ (LTE_4_) excretion. Following sputum induction and determination of cellular inflammatory pattern based on sputum cells differential count, NEA was defined as neutrophilic or paucigranulocytic.^6^ Induced sputum supernatant was measured for proinflammatory mediators: prostaglandin D_2_ (PGD_2_), leukotriene E4 (LTE_4_) and anti‐inflammatory prostaglandin E_2_ (PGE_2_). CRS comorbidity was scored by computed tomography of the paranasal sinuses using Lund‐Mackay scale by two experienced radiologists.

### 2.3 Follow‐up examination

Exacerbations during the year preceding the study were defined as asthma symptoms requiring hospitalization or systemic steroid therapy. Asthma severity was assessed retrospectively based on the level of treatment required to control the patient's symptoms and exacerbations. Severe asthma was defined as uncontrolled asthma despite optimized treatment with high‐dose ICSs and long‐acting β_2_‐agonists, or asthma that required high‐dose ICS and long‐acting β_2_‐agonists to prevent becoming uncontrolled. Moderate asthma was defined as well controlled asthma using Step 3 treatment.^15^ Mild asthma was defined as well controlled asthma using ICS‐ formoterol as needed or with a low dose ICS alone.^15^

### 2.4 Induced sputum collection and inflammatory phenotype based on sputum cells

Induced sputum was collected according to the European Respiratory Society recommendations.^16^ Details were described elsewhere.^2^, ^3^, ^17^ Four inflammatory patterns based on sputum cells were identified: eosinophilic (≥3% eosinophils and <64% neutrophils), neutrophilic (≥64% neutrophils and <3% eosinophils), mixed (≥3% eosinophils and ≥60% neutrophils), and paucigranulocytic (<3% eosinophils and <64% neutrophils). The mixed phenotype was considered transient between eosinophilic and neutrophilic phenotypes and was not included in this classification.^6^

### 2.5 Sputum eicosanoids

Sputum eicosanoid levels were measured by gas chromatography and tandem mass spectrometry for PGD_2_ and PGE_2_ or by high‐performance liquid chromatography and tandem mass spectrometry for LTE_4_. Results were expressed in picograms per milliliter. Analytical details were described elsewhere.^3^, ^17^, ^18^

### 2.6 Urinary leukotriene E_4_

Urinary samples were collected in the morning, and an enzyme‐linked immunosorbent assay (Cayman Chemical Co., Ann Arbor, MI, USA) was used to assess urinary LTE_4_ excretion. Results were presented in picograms per milligram of urinary creatinine.

### 2.7 Statistical analysis

Data were analyzed using Statistica 13.1 software package (TIBCO Software, Palo Alto, CA, USA) or R package v.4.1 (The R Foundation for Statistical Computing Platform 2021, University of Vienna, Austria). Descriptive statistics for demographic, clinical, and laboratory characteristics were presented as median with 25th and 75th percentiles for continuous variables or as a frequency for categorical variables. The analysis of variance with post‐hoc Tukey procedure was used to compare continuous variables between clusters. The Spearman rank correlation coefficients were used to assess the associations between quantitative variables. A *p* value of less than 0.05 was considered significant.

### 2.8 Clustering strategy and cluster validation

Detailed information about cluster analysis is described in an Online Supplement.

A hierarchical cluster analysis based on 16 variables covering 5 qualitative ones: sex, skin tests, asthma control, asthma severity, inflammatory phenotype, and 11 quantitative variables: body mass index, age, age at asthma onset, exacerbations, blood eosinophil counts, total serum IgE, FEV_1_, urinary LTE_4_, sputum LTE_4_, PGD_2_, and PGE_2_. Internal cluster validation using different indices was performed to evaluate clustering propensity. One of these indices, the silhouette width (S_i_), quantified each point in one cluster by a distance to the points in the neighboring clusters. By this definition, S_i_ close to 1 was expected for very well clustered data, whereas S_i_ close to 0 characterized a case located between two clusters. In a hierarchical cluster tree, the height axis indicated dissimilarity between 2 clusters, with increasing height for the less similar the objects. Because this height represented a cophenetic distance between two objects, to assess how the cophenetic distances reflected the original distances, a correlation coefficient was calculated between the two distances.

## 3 RESULTS

**FIGURE 1:**
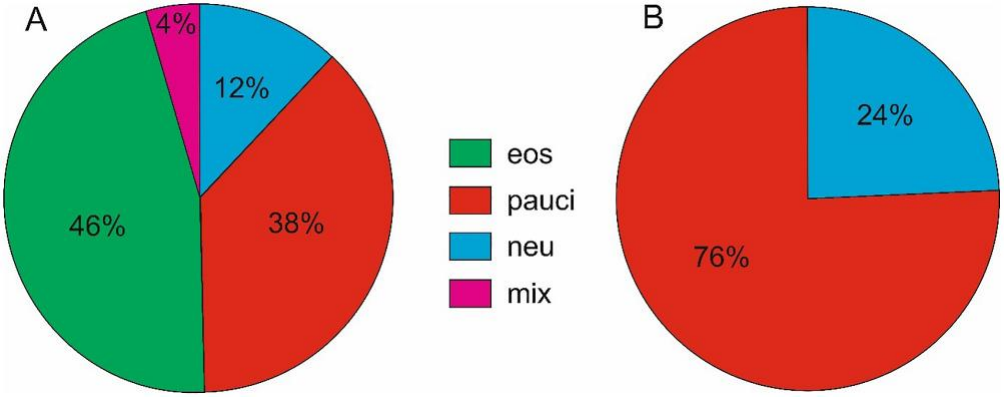
(A) Distribution of patients with nonsteroidal anti‐inflammatory drug–exacerbated respiratory disease (N‐ERD) (*n* = 133) according to the cellular inflammatory pattern of the induced sputum. Paucigranulocytic (38%) and neutrophilic (18%) phenotype was considered as non‐eosinophilic asthma (NEA), and these patients (*n* = 66) were included in the study. (B) Distribution of patients with paucigranulocytic or neutrophilic asthma phenotype among NEA patients with N‐ERD. eos, eosinophilic; mix, mixed; neu, neutrophilic; pauci, paucigranulocytic.

The distribution of patients according to cellular inflammatory patterns on all N‐ERD and NEA subgroup is presented in Figure 1AB. NEA was present in 66 N‐ERD patients (49.6%) and was stratified into neutrophilic or paucigranulocytic inflammatory pattern.^6^ Paucigranulocytic asthma (PGA) was identified in 50 (75.8%) patients of the N‐ERD subgroup with NEA, whereas 16 (24.2%) patients had neutrophilic asthma (NA). The mean induced sputum macrophage fraction of patients with PGA phenotype was significantly higher than in patients with the neutrophilic one (36.9% vs. 13.1%; *p* = 0.006). Following the clustering analysis, four candidate clusters of NEA were distinguished among N‐ERD patients with non‐eosinophilic asthma. Cophenetic correlation for the final cluster tree was *r* = 0.47. The demographics and clinical characteristics of patients in each cluster are shown in Table 1. A dendrogram showing a hierarchical clusters of the studied NEA patients with N‐ERD is presented in Supplemental Figure 1.

### 3.1 Characteristics of non‐eosinophilic N‐ERD clusters

The largest cluster #3 included 25 N‐ERD patients (37.9%), the most of whom had PGA (96%). Clinically, patients within this cluster had severe asthma (96%) and were older than those in clusters 2 and 1. Moreover, their age at the onset of asthma was higher and body mass index was greater than in the cluster #2 (Table 1). There were no significant differences in the rate of asthma exacerbations in the previous year compared with the other clusters (Table 1). Prevalence of neutrophilic pattern was the lowest in this cluster in comparison with all the remaining clusters combined (4% vs. 37%, *p* = 0.003). Induced sputum level of PGD_2_ was a biomarker of cluster #3 because it was the lowest. Cluster #2 comprised 17 patients (25.8%) with mild (52.9%) or severe (47.1%) non‐eosinophilic inflammatory pattern, mainly PGA (76.5%). A common clinical feature of these N‐ERD patients was well‐controlled asthma reported in 94.1%. No asthma exacerbation in the previous year was noted for 70.6% of patients in this cluster versus 25% of patients in cluster #1 (*p* = 0.015). Moreover, these patients had a significantly lower age of asthma onset versus patients in cluster #3. There were no significant differences in the percentage distribution of PGA and NA phenotypes between cluster #2 and the remaining clusters. Cluster #1 included 16 N‐ERD patients (24.2%) with NEA. They had equal distribution of PGA and NA. All patients had severe asthma, and their level of asthma severity was significantly higher than in clusters #2 and #4. Asthma exacerbations in the year preceding the study were noted in 75% of patients in this cluster. Patients in cluster #1 had significantly lower blood eosinophil count than patients in clusters #2 and #4, they had also a significantly lower total serum IgE level than patients in cluster #4. Induced sputum level of PGD_2_ was the highest by comparison with other clusters. Elevated PGE_2_ sputum levels were also observed, when compared to patients in clusters #2 and #3. However, sputum prostaglandins did not correlate with cellular inflammatory pattern of the sputum in neither cluster. The smallest cluster #4 included 8 N‐ERD patients (12.1%) with moderate and well‐controlled NEA. There was insignificant predominance of PGA (65.5%). The average level of asthma severity was significantly lower in cluster #4 and asthma control was significantly better than in clusters #1 and #3. However, in half of cluster #4 patients exacerbations were noted in the previous year. Moreover, patients in this cluster had significantly higher blood eosinophil count and serum total IgE levels than these in cluster #1. They had also significantly lower sputum PGD_2_ levels than these in cluster #1. Therefore, some significant differences in the distribution of cellular inflammatory patterns were noted only between cluster #3 (PGA) and clusters #1 or #4.

### 3.2 PGD_2_ as a discriminatory biomarker of non‐eosinophilic N‐ERD clusters

**FIGURE 2:**
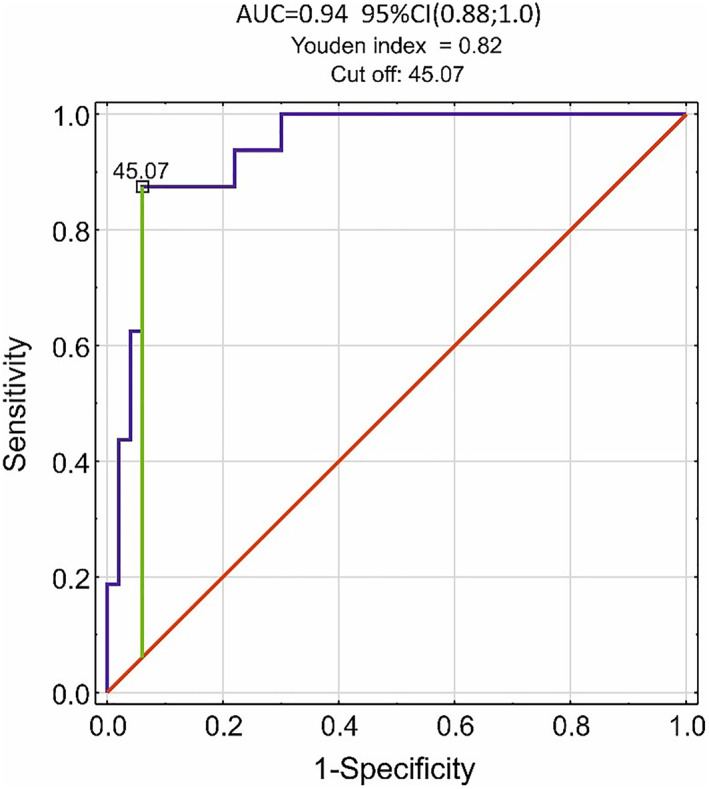
A receiver operating characteristic analysis of non‐eosinophilic asthma clusters using sputum prostaglandin D_2_ to distinguish between cluster #1 and clusters #2, #3, and #4. AUC, area under the curve; CI, confidence interval.

**FIGURE 3:**
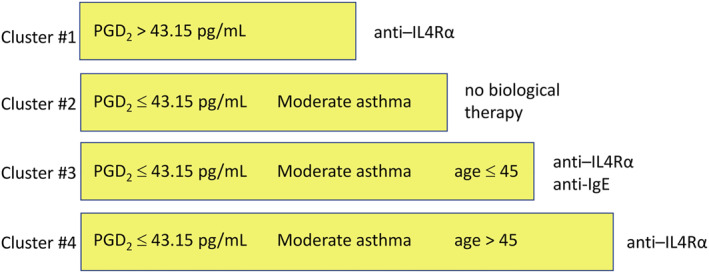
A simplified decision tree for assignment of the study subjects into subtypes of N‐ERD with non‐eosinophilic asthma using only 3 variables (sputum PGD_2_ level, severity of asthma, and age of the patient). An available biological therapy is proposed on the premises of tentative pathomechanism underlying the clusters.

Decision tree analysis confirmed the key role of sputum PGD_2_ levels on clustering results. PGD_2_ levels were the highest in cluster #1 patients, with an area under the receiver operating characteristic curve (AUROC) of 0.94 (95% CI, 0.88, 1.0). Using the Youden cut‐off value of PGD_2_ = 45.07 pg/ml, accuracy of the discrimination of cluster 1 member was 92.4% (95% CI, 83.2%–97.5%), sensitivity = 87.5% (95% CI, 61.7%–98.5%) and specificity = 94.0% (95% CI, 83.5%–98.8%). In the studied NEA group of N‐ERD patients, sputum PGD_2_ level had positive predictive value for cluster #1 membership PPV = 82.45% (95% CI, 60.5%–93.4%), whereas negative predictive value was NPV = 92% (95% CI, 86.5%–98.9%; Figure 2). Sputum PGE_2_ levels were also significantly higher in cluster #1 but this biomarker had a worse predictive value. No association was present between eicosanoids in sputum and disease severity. Urinary excretion of LTE_4_, although elevated in cluster #1, had no biomarker properties for assignment of patients to any cluster. There was a good agreement (Cohen's kappa = 0.79) between the decision tree rules and cluster analysis. Therefore, the list of predictors of non‐eosinophilic N‐ERD variants could be limited to the age of patients, their severity of asthma and to the sputum level of PGD_2_ (Figure 3). In summary, cluster #1 included patients with sputum PGD_2_ levels exceeding 43.15 pg/ml, cluster #4 was represented by patients with sputum PGD_2_ levels less or equal to 43.15 pg/ml and moderate asthma, cluster #2 by sputum PGD_2_ levels ≤43.15 pg/ml and mild or severe asthma, and age ≤45 years, whereas cluster #3 comprised patients with sputum PGD_2_ levels ≤43.15 pg/ml, mild or severe asthma, and age >45 years.

## 4 DISCUSSION

Eosinophilic inflammation of airways is considered a characteristic feature of N‐ERD, but numerous patients with the disease have also a non‐eosinophilic cellular inflammatory pattern^2^, ^3^, ^4^, ^17^ Research in N‐ERD was focused on eosinophilic inflammation, thus data on NEA in this particular phenotype of asthma is limited. Using widely accepted definition of cytological findings in the sputum, two non‐eosinophilic cellular patterns in N‐ERD are paucigranulocytic and neutrophilic. In a prospective N‐ERD cohort of this study, almost half of patients had NEA, a predominant pattern was PGA. Therefore, eosinophilic mucosal inflammation, although frequent in allergic asthma, is not specific for asthma patients with N‐ERD. A similar observation was recently suggested by a study of CRSwNP endotypes in N‐ERD, based on nasal mucus biomarkers.^12^ Important consequence of such heterogeneity is a limited efficacy of highly effective biologic treatment in N‐ERD, for example, mepolizumab targeting IL‐5.^14^ It is tempting to re‐evaluate the role of eosinophil in N‐ERD. These cell is an important source of inflammatory mediators during an acute reaction to NSAIDs, but perhaps not mandatory to maintain intolerance to COX‐1 inhibitors. Importantly, eosinophils collected from the respiratory tract of patients with N‐ERD demonstrated elevated expression of LTC_4_ synthase and produced IFN‐γ along with PGD_2_, release of which was stimulated by NSAIDs.^19^ Patients with N‐ERD had reduced levels IL‐5 and IL‐13 in polyps, whereas these type 2 cytokines were characterizing T2‐high hyperplastic eosinophilic CRSwNP. Moreover, IL‐4 and IFN‐γ levels were reported increased in N‐ERD with CRSwNP.^19^ However, in another study by Stevens et al.^21^ no differences were observed between N‐ERD and CRSwNP without aspirin intolerance in IFN‐γ, IL‐5, and IL‐13 polyp tissue levels. Eosinophilic inflammation in N‐ERD was suggested to result from non‐Th2 mediators activity, rather than classical type 2 cytokines and related chemoattractants.^20^ To explain this unorthodox pathomechanism of N‐ERD, cellular or transcellular mediators were proposed such as produced by granulocyte‐platelet aggregates,^21^ tissue resident mast cells,^22^ innate lymphoid cells,^23^ basophils,^24^ or macrophages.^25^ Cytological classification based on infiltrating respiratory tissue granulocytes revealed that in 30% N‐ERD patients a mixed inflammation was present, also with some neutrophilic inflammatory pattern alone.^26^

In the current study, the highest number of N‐ERD patients without eosinophilic inflammatory pattern had severe asthma and were characterized by the paucicellular sputum cytology (cluster #3). Our results indicate that macrophages are predominant in the sputum of patients with PGA phenotype, in line with a study by Olgac et al.^27^ But in contrast to alveolar macrophages of healthy subjects, a recent study demonstrated that macrophages can be reprogrammed epigenetically in N‐ERD and possibly promoted to type 2 inflammation. Alveolar‐like monocyte‐derived macrophages collected from N‐ERD patients produced more 5‐lipoxygenase–derived proinflammatory lipid mediators than in healthy controls.^25^ In response to a proinflammatory stimulation with bacterial lipopolysaccharide, these macrophages upregulated expression of CXCL2 and CXCL8, neutrophilic chemokines and non‐specific proinflammatory cytokines IL‐1β and TNFα. It was also suggested that a persistent reprogramming of macrophages underlain chronic type 2 inflammation.^25^ However, inflammatory cellular and cytokine patterns in N‐ERD are broad, encompassing both Th2‐high and Th2‐low inflammation. PGA is generally a stable disease and the one most frequently treated with high doses of ICSs, which could mask initial inflammatory pattern present before the onset of treatment.^28^ In the majority of asthmatics who had conversion of cellular inflammatory pattern after the first month of fixed ICS treatment, PGA changed to NA.^29^ We suggest that PGA is an important inflammatory pattern in severe N‐ERD. Patients who aggregated into cluster #3 had lower sputum PGD_2_ and PGE_2_ levels than these in cluster #1 representing also a severe N‐ERD. A therapeutic intervention using biologics targeting both eosinophilic asthma and NEA, like anti‐thymic stromal lymphopoietin antibodies^30^, ^31^, ^32^ or anti‐IL‐4Rα could be beneficial for this N‐ERD subset. In fact inhibition of IL‐4Rα decreasing both IL‐4 and IL‐13 signaling, was associated with the greatest improvement across N‐ERD patients.^14^ Cluster #1 also included N‐ERD patients with severe NEA. Compared with cluster #3, a higher percentage of patients in cluster #1 had NA inflammatory pattern. Moreover, patients in cluster #1 had the highest PGD_2_ levels in sputum suggesting increased cyclooxygenases expression. Results of PGD_2_ receptor antagonists trials in asthma could change if were conducted in N‐ERD patients displaying cluster #1 characteristics. Especially, dual D‐prostanoid (DP_1_) and chemoattractant receptor‐homologous molecule expressed on Th2 cells (CRTH2/DP_2_) antagonists, promising in animal models of inflammatory diseases, decreased macrophage activation and neutrophil accumulation.^33^, ^34^ PGD_2_ was shown to significantly augment inflammation by its ability to enhance vascular permeability, the proinflammatory action of macrophages and subsequent neutrophil migration and activation.^35^ It can be only speculated, whether blockade of DP_1_ along with DP_2_ would be sufficient to abolish PGD_2_‐induced activation of macrophages, since a downstream metabolite of PGD_2_ activates also proinflammatory signaling by the receptor for thromboxane (TP).^35^ However, no dual anti‐PGD_2_ drugs have been approved for clinical use yet. Recently Kermani et al.^36^ reported about inflammasome activity in patients with severe asthma and neutrophilic inflammatory pattern. Again, anti‐inflammasome intervention is theoretically possible in such endotype of asthma.^6^ The remaining non‐eosinophilic N‐ERD patients were characterized by a well‐controlled asthma. In cluster #2, non‐eosinophilic cellular inflammatory pattern was not specific, and there was no history of disease exacerbations. But these patients had the youngest age of asthma onset. Apparently, patients with a mild disease in cluster #2 did not require biological treatment, despite a similar burden of CRSwNP as in other subgroups. The European Academy of Allergy and Clinical Immunology Asthma guidelines included recommendations for comorbid CRSwNP.^37^ In cluster #4 all the patients were atopic, had an increased blood eosinophil count and CRSwNP burden. It is possible, that this small group of N‐ERD patients corresponded to a classical presentation of N‐ERD, but with an excellent response to ICS. Their non‐eosinophilic inflammatory pattern in sputum could result from conversion induced by ICS into PGA or NA. According to GINA^15^ guidelines, these patients did not require biological therapy of asthma.

In this study we identified four subtypes of N‐ERD which departed from a classical eosinophilic asthma characteristics. Distinction of this subtypes was made using basic clinical data and lipid inflammatory mediators in sputum. We propose a decision tree stratification of non‐eosinophilic N‐ERD, assigning patients into clusters using only 3 variables (age of a patient, asthma severity and induced sputum PGD_2_ level). Discrimination power of sputum PGD_2_ levels seems critical for a correct stratification of non‐eosinophilic N‐ERD. Because of complex methodology for sputum PGD_2_ measurements, a more readily available assays of PGD_2_ metabolites in urine have to be validated for this purpose in a future research. Acknowledging expansion of biologics trials in asthma and their limited efficacy in N‐ERD patients, we summarize potential interventions in severe non‐eosinophilic N‐ERD using mechanistic premises (Figure 3).

Several limitations of the current study have to be listed. First, some bias due to a small study group was inevitable despite a rigorous statistics employed. Second, all the patients of the study received anti‐asthmatic treatment for the same period of 52 weeks, which was topical corticosteroids and long‐acting β_2_ agonists for at least 6 weeks prior induction of the sputum. By this approach, complying with the study ethics, no stability of cellular inflammatory pattern in the sputum was ensured. Especially patients in cluster #4 could represent a beneficial effect of ICS, whilst their initial cellular pattern of inflammation could be eosinophilic. Third, this was a single center study conducted in a tertiary reference clinics, thus the study group was not representative for a real‐life N‐NERD patients.

In conclusion, asthma patients with N‐ERD have extensive inflammation of airways, which is either eosinophilic or non‐eosinophilic. We focused on non‐eosinophilic inflammatory pattern identified by the induced sputum cytology of N‐ERD to describe its heterogeneity. We identified subtypes of non‐eosinophilic N‐ERD as distinguished by clinical features and a single biomarker, that is, sputum PGD_2_ level. Results of this study confirms involvement of inflammatory cells other than eosinophils, namely neutrophils and macrophages in the pathogenesis of N‐ERD. Because successful trials of novel therapies for N‐ERD seem dependent on a definition of a precise endotype of the disease,^38^ future research should focus on identification of biomarkers of heterogeneity feasible to measure in clinical practice.

## AUTHOR CONTRIBUTION

L. M. and M. S. involved in conception or design of the work. L.M., N.C.W., J.S., K.H.‐S. and J.T. collected the data. L.M., A.C., KW., and M.S. were involved in data analysis and interpretation. L.M. drafted the article. M.S. critically revised the final version of the manuscript. All authors provided critical feedback and helped shape the research, analysis, and manuscript.

## CONFLICT OF INTEREST STATEMENT

None declared.

## Supporting information

Supplementary Material

## Data Availability

The data that support the findings of this study are available from the corresponding author upon reasonable request.
