# Supplementary material for: Non‐eosinophilic asthma in nonsteroidal anti‐inflammatory drug exacerbated respiratory disease
Source: Clin Transl Allergy. 2023 Mar 13;13(3):e12235. doi: 10.1002/clt2.12235 (PMC10009799; doi:10.1002/clt2.12235)
Supplement: Supplementary file 1 — Supplementary Material [file CLT2-13-e12235-s001.docx]

**ONLINE SUPPLEMENT**

**Non-eosinophilic asthma in nonsteroidal anti-inflammatory drug–exacerbated respiratory disease**

**Clustering strategy**

To identify groups of similar cases, a cluster analysis was performed using the following R packages: FactoMineR, factoextra,^1,2^ NbClust,^3^ fpc, and pvClust.^4^ The data matrix comprised 66 cases and 16 variables (5 quantitative and 11 qualitative ones). Clustering tendency was assessed using the Hopkins statistic (H) and visual assessment of cluster tendency algorithm^5^ to confirm a non-random structure of clusters. To reduce dimensionality, a factor analysis of mixed data (FAMD) was used, allowing for both quantitative and qualitative variables input. Thus raw variables of the data matrix were subsided by 8 continuous principal components explaining in total 72.3% of the variability. FAMD is considered as a denoising procedure, which allows for a more stable clustering. Unsupervised hierarchical cluster analysis was done using the Ward’s algorithm based on Euclidean distance. To determine the optimal number of clusters, the NbClust function in the R package was used. In this method, 30 available indices were computed, including Elbow, CCC, Silhouette and Gap statistics, to select the most appropriate number of clusters based on the majority rule. Internal validation of clusters was performed without reference to external information on assignment of cases. Computed indices of the cases grouping into four clusters measured compactness, connectedness, and separation of the cluster partition. Next, a decision tree analysis was performed using all 16 raw variables to predict the cluster assignment for each data case. Agreement between clustering and decision tree grouping was calculated as Cohen's kappa coefficient, which evaluates inter-ratter reliability.

**
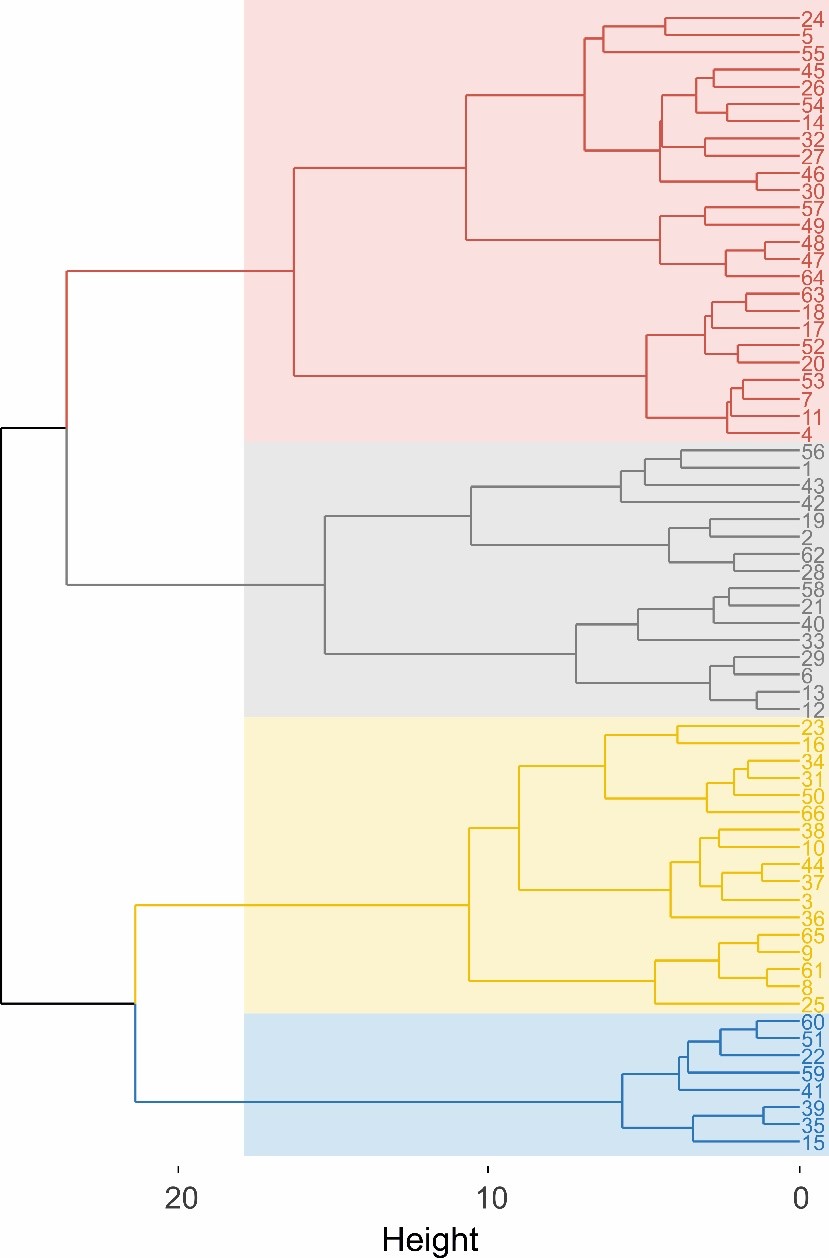
**

**Supplemental Figure 1.** A dendrogram showing a hierarchical cluster analysis of 16 variables among the 66 patients with nonsteroidal anti-inflammatory drug–exacerbated respiratory disease and non-eosinophilic asthma. Cluster analysis was performed using the Ward method on Spearman's correlation. Each color represents a different cluster: blue – cluster #4, yellow – cluster #2, gray – cluster #1, red – cluster #3.

**Correlations between studied biomarkers**

The percent of sputum neutrophils (%) and determining neutrophilic inflammatory pattern did not correlate with PGD_2_ levels in the induced sputum supernatant in the whole NEA group (n = 66). The correlation re-analysed in clustered subsets were also insignificant. Only in cluster #1, there was a weak trend for a negative correlation between sputum neutrophils (%) and PGD_2_ levels (r = -0.435; *P*=0.09).

**References**

1 Kassambara A. Practical Guide to Cluster Analysis in R. Unsupervised Machine Learning. Multivariate Analysis I, STHDA. 2017. <http://www.sthda.com>

2 Kassambara A. Practical Guide to Principal Component Methods in R Multivariate Analysis II, STHDA. <http://www.sthda.com>

3 Charrad M, Ghazzali N, Boiteau V, Niknafs A NbClust: An R Package for Determining the Relevant Number of Clusters in a Data Set. *J Stat Softw.* 2014;61:1-36. <http://www.jstatsoft.org/v61/i06/>

4 Ryota S, Hidetoshi S. Pvclust: an R package for assessing the uncertainty in hierarchical clustering. *Bioinformatics*. 2006;22:1540-1542. <https://doi.org/10.1093/bioinformatics/btl117>

5 Bezdek J, Hathaway RJ. VAT: A tool for visual assessment of (cluster) tendency. Proceedings of the International Joint Conference on Neural Networks. 2002. <https://ieeexplore.ieee.org/xpl/conhome/7877/proceeding>
